# Supplementary material for: On the equilibrium limit of liquid stability in pressurized aqueous systems
Source: Nat Commun. 2024 Dec 18;15:10666. doi: 10.1038/s41467-024-54625-z (PMC11655544; doi:10.1038/s41467-024-54625-z)
Supplement: Supplementary file 1 — Supplementary Information [file 41467_2024_54625_MOESM1_ESM.pdf]

Supplementary Information

On the equilibrium limit of liquid stability in pressurized aqueous systems

Arian Zarriz[1], Baptiste Journaux[2]✉, Matthew J. Powell-Palm[1,3,4]✉

[1]J. Mike Walker ’66 Department of Mechanical Engineering, Texas A&M University, College Station, TX, USA

[2]Department of Department of Earth and Space Sciences, University of Washington, Seattle, WA, USA

[3]Department of Materials Science & Engineering, Texas A&M University, College Station, TX, USA

[4]Department of Biomedical Engineering, Texas A&M University, College Station, TX, USA

Correspondence:

BJ (bjournau@uw.edu)

MPP (powellpalm@tamu.edu)

This PDF file includes:

Supplementary Notes S1-S5

Supplementary Figures S1 to S6

Supplementary Table S1

Supplementary References

Supplementary Note 1. Isochoric Freezing and Melting Methodology

The isochoric freezing approach employed here is detailed in the main text and in Chang et al. (main text reference 1), but for the aid of the reader seeking to employ isochoric freezing, we will provide further commentary on the methodology herein.

Isochoric freezing and melting is a thermo-volumetric method used to investigate the phase transitions and equilibria of aqueous solutions. By constraining the volume and concentration of a eutectic binary solution, per Gibbs Phase Rule, the system is forced into a 1-DoF thermodynamic configuration, wherein its equilibrium is fully prescribed by one intensive variable. In practice, the experimentalist controls the temperature, allowing the pressure to respond per Le Chatelier's principle and recording it.

For the binary solutions studied here, the isochoric freezing and melting process proceeds as follows. First, a solution at the 0.1 MPa eutectic concentration is prepared. We used the literature values of atmospheric eutectic concentration listed in Table S1. We note that experimental uncertainty in the initial concentration of the solution will affect the relative phase fractions of the phases present in the eutectic assemblage, but will not substantially affect the P-T trajectory of the equilibrium curve, because there is only one intensive thermodynamic degree of freedom.

This liquid is then loaded (absent any air bubbles) into a rigid pressure-bearing Al7075 isochoric chamber (internal volume 5.33 mL) equipped with an ESI GD4200-USB-4000-DE digital pressure transducer (Figs. S1.a and S1.b). These transducers enable high-fidelity pressure measurements up to 400 MPa, at a sampling frequency of 1 Hz. They are operated via a proprietary "ESI-USB" data acquisition software available from the company.

Upon filling the chamber, the pressure transducer is threaded into the chamber and torqued down to 45 N.m. This torque value ensures effective chamber closure via metal-on-metal surface sealing (as labeled in Figure S1.b), while preventing undue stress on the sensor housing.

Supplementary Table 1. Atmospheric eutectic concentration for different binary solutions

Solute	Eutectic concentration at 0.1 MPa (wt%)	Reference
**Na_2CO_3**	5.88	*Pascual, M., et al.*[1]
**KCl**	19.50	*Li, Gang, et al.*[2]
**$MgSO_4$**	17.30	*Pillay, Venasan, et al.*[3]
**Urea**	32.80	*Yuan, Lina, et al.*[4]
**Na_2SO_4**	4.15	*González Díaz, C., et al.*[5]
**NaCl**	23.30	*Journaux et al., Drebushchak et al.*[6]
**$NaHCO_3$**	6.15	*Pascual, M., et al.*[1]
**$MgCl_2$**	21.60	*Ketcham, S. A. et al.*[7]*, González Díaz, C., et al.*[5]

To maintain controlled temperature conditions for the electronics housed within the pressure transducers during the experiment (independent of the temperature of the chamber/sample), a 13W polyimide film heater with a 70 mm diameter is affixed to each pressure sensor. These heaters are regulated by a simple thermal relay responding to the observed temperature of the transducer's electronics housing, and they are set to maintain a constant temperature of 306.15 ± 0.5K. The relays are powered by a generic 12V DC power supply.

A 3D-printed lid was subsequently constructed to enable mounting of three chambers simultaneously in the bath, facilitating both total immersion of the isochoric chambers within the cooling fluid and insulation of the pressure transducer electronics housing above (Figure S1.d).

Two cooling baths were employed, as described in the main text. A PolyScience AP15R-40 bath was employed for binary solutions with ice-III transition temperatures higher than 243.15K, while a 7380 Fluke ultra-low temperature bath was utilized to achieve lower temperatures, reaching as low as 193.15K. This setup enabled high-throughput data collection for various binary solutions.

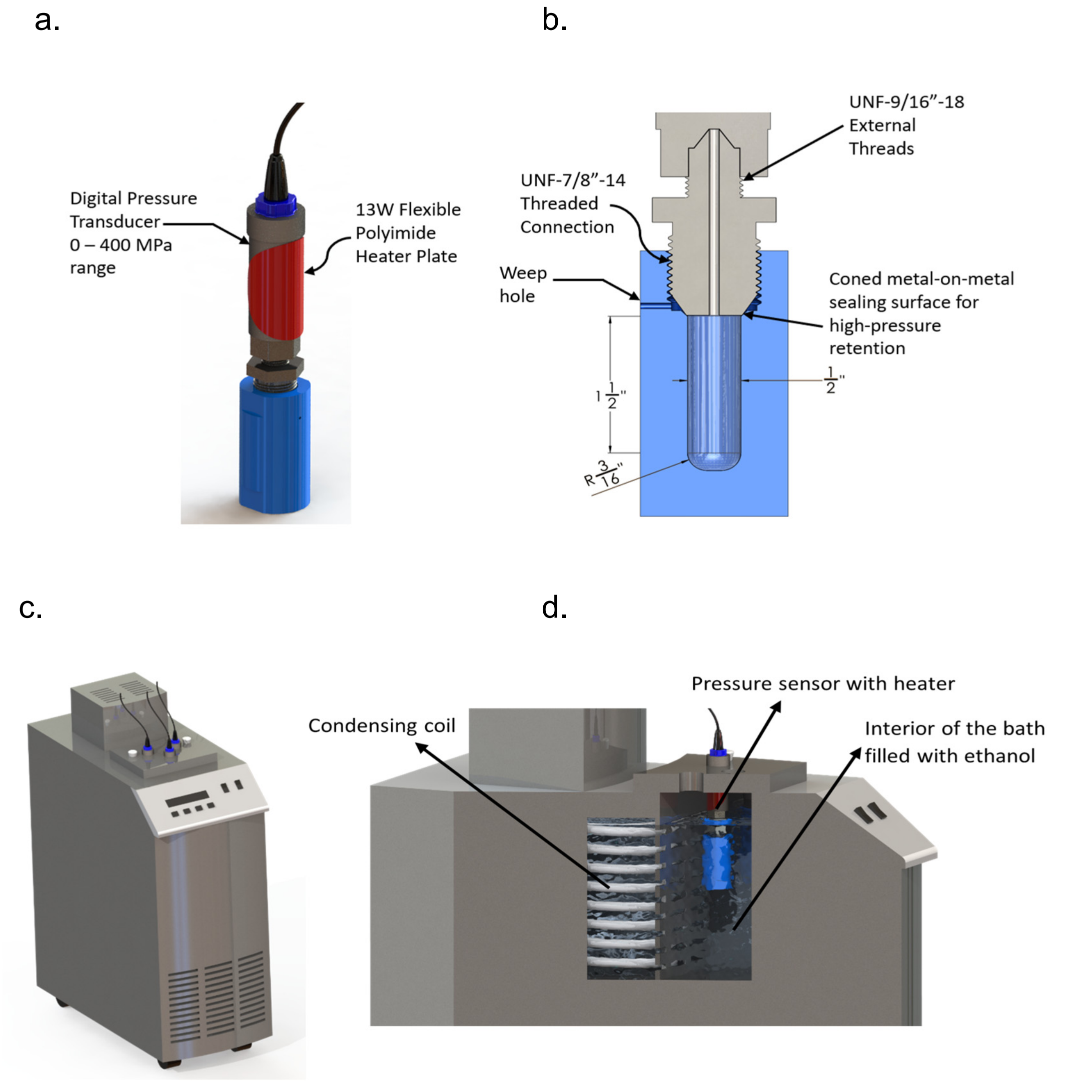

Supplementary Figure 1: Isochoric freezing and melting assembly. a) 3D render of Al7075 isochoric chamber (blue) and pressure transducer assembly, b) 2D cross-section of isochoric chamber and pressure transducer assembly, c, d) 3D renders of the fully assembled cooling bath (Fluke), chamber, and pressure transducer assembly.

Cooling and warming processes were programmed using on-board software for the PolyScience bath, and via MATLAB for the Fluke bath. The temperature within the bath was also recorded using a calibration-grade Fluke stick thermometer, which was submerged in the bath near the chambers to ensure precise temperature measurement.

The PolyScience AP15R-40 bath was filled with a solution of water and 60% w/w ethylene glycol, in a total volume of 15 liters, per manufacturer specifications. The manufacturer-rated temperature accuracy of this bath at steady state is 0.005 °C. The large volume and high viscosity of this coolant provided a relatively high thermal resistance and relatively low cooling power to the chambers, necessitating 45 minute warming steps per 0.5K warming increment to guarantee steady-state pressure (<0.1

MPa/min). We note that this cooling bath and warming time step are identical to those employed by Chang et al. for the same protocol.

The Fluke bath was filled with a solution of water and 95% w/w ethanol, per manufacturer specifications. The manufacturer-rated temperature accuracy of this bath at steady state is 0.005 °C. The lower solution viscosity and volume as compared to the PolyScience bath provided considerably higher cooling power and faster heat transfer, necessitating 20 minute steps per 0.5K warming increment to guarantee steady-state pressure (<0.1 MPa/min).

Supplementary Note 2. Example time-series pressure-temperature data

In order to obtain equilibrium data with the highest degree of confidence possible, the P-T coordinates reported in the main text were acquired upon slow warming of the solutions. However, the general phenomenology of the cooling process may be of interest to the reader seeking to perform isochoric experiments, and we thus provide a typical cooling and warming profile below.

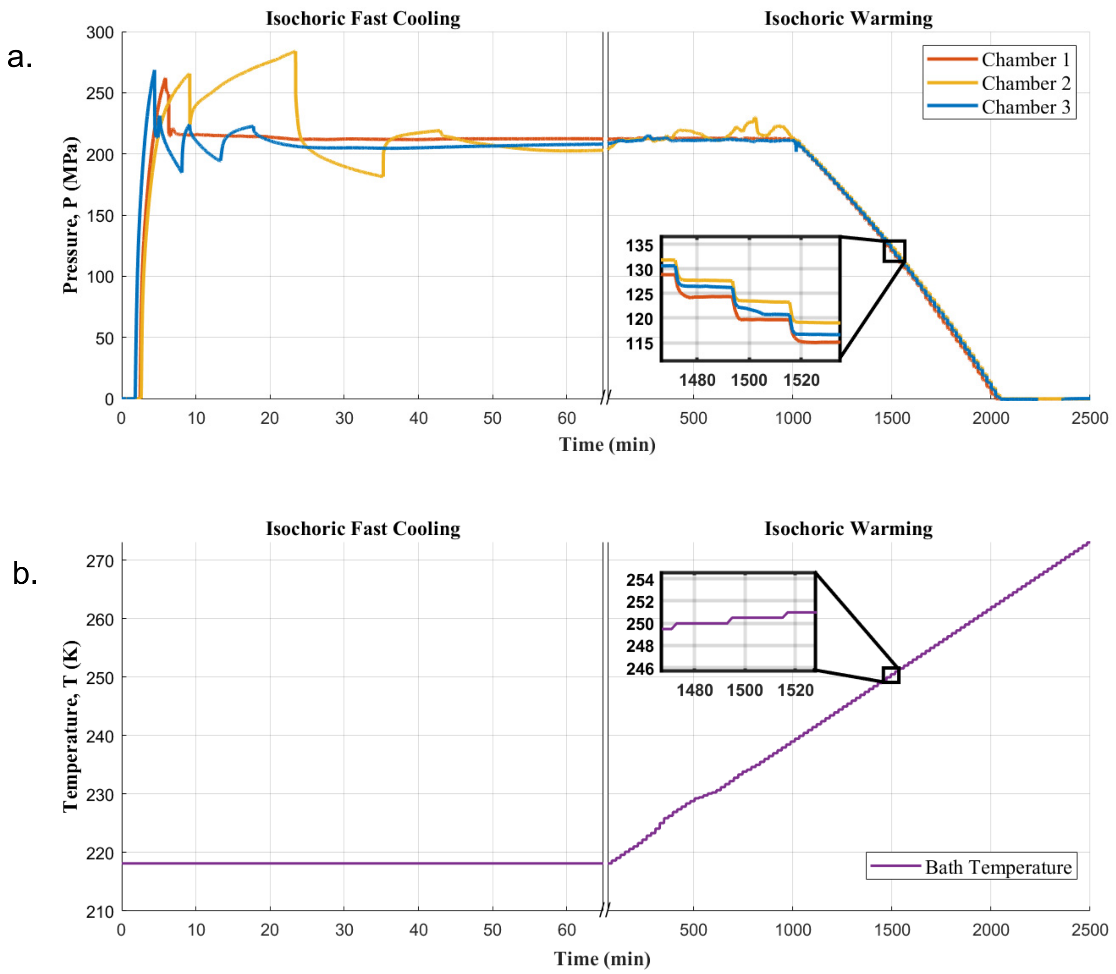

Supplementary Figure 2. Sample fast cooling and standard warming protocol for KCl. a) Pressure-Time profile in three chambers under fast cooling protocol and slow warming with 0.5K per 20 minutes (Fluke bath). b) Measured Temperature-Time profile in the same.

During the fast-cooling process depicted in Figure S2, with the bath set to 218.15K, two phase transition events are observed. In the first five minutes of cooling, the chamber pressures increase rapidly, which is compatible with growth of larger-volume ice Ih. It notably overshoots the ice Ih-II solid-solid phase transition around 210 MPa, producing a metastable ice-Ih liquid brine assemblage. Subsequently, we observe large drops in pressure followed by strong pressure fluctuations suggesting the formation of denser phases. Considering the pressure and temperature, it is reasonable to associate these variations with the formation of dense KCl salt and/or ice II for pressure drops, and ice Ih for pressure increases, until reaching an equilibrium assemblage. The significant variations between each chamber in the first 40 minutes of cooling also suggest strong hysteresis expected for out of equilibria conditions typical for rapid cooling of aqueous solutions. The phase configuration stabilizes at approximately 209 MPa for each chamber. The final transition characteristics of pressure and temperature align with the pressure and temperature range of the ice-II region in the water phase diagram.

During the warming process we observe small pressure fluctuations that could correspond to formation of ice Ih from ice II or ice III, until the assemblage reaches starts to produce melt, as marked by a 'kink' in the pressure curve around the 1000 minute mark. This point marks the unique "cenotectic" point for each solution, after which the pressure decreases monotonically as the temperature increases, following the eutectic curve along the brine-ice Ih-KCl equilibrium line. Each step in this process takes 20 minutes, with approximately 2 of these minutes required to stabilize the temperature within the bath, which is increased in 0.5K increments. This stabilization period was chosen to ensure equilibration of each chamber sample with the surrounding bath temperature, which is confirmed by the stabilization of the pressure signal (as shown in the insets of Fig S2).

The temperature at which the system reaches atmospheric pressure corresponds to the eutectic temperature of the solution.

Supplementary Note 3: Additional data on solutions with apparent intermediate hydrates

For more details on P-T profiles in solutions with intermediate hydrates, Figures S3 - S5 below show every run recorded for the binary solutions found to produce intermediate high-pressure hydrate phases. General conclusions about the data shown here do not deviate from those presented in the main text, but we suspect that these additional data may be of interest to researchers studying any one of these solutions in greater depth.

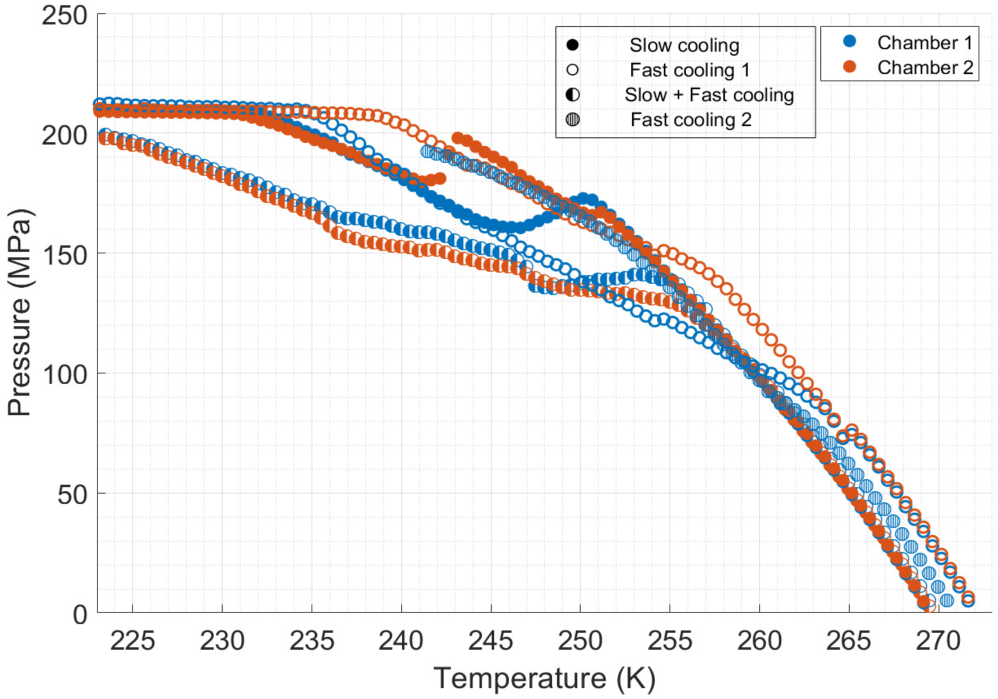

Supplementary Figure 3. All individual P-T curves recorded for NaHCO3.

NaHCO3 exhibited the greatest diversity of P-T transition features of the solutions considered, and as such, we experimented with additional cooling protocols in an effort to solicit more behaviors. In addition to the slow cooling and slow+fast cooling protocols described in the main text, we also tried two fast cooling protocols:

Fast Cooling 1: Submersion directly from room temperature to 213.15K.

Fast Cooling 2: Submersion directly from room temperature to 241.15K.

In Figure S3 above, it should be noted that the metastable phase configurations and byproducts produced during fast cooling appear to linger to higher temperatures than those for slow cooling. For these protocols, metastable byproducts appear to linger all the way to atmospheric pressure, affecting the recorded atmospheric pressure eutectic point, with greater deviation in eutectic temperature measured for greater cooling rate. While this phenomenon needs significantly more investigation in order to draw any firm conclusions, this behavior suggests that there *may* be metastable hydrates of NaHCO3 that can persist at atmospheric temperature at around 273.15 K. These intermediate phases warrant further investigation, especially via metrological means that may produce more granular information on hydrate structure, such as x-ray diffraction or Raman spectroscopy.

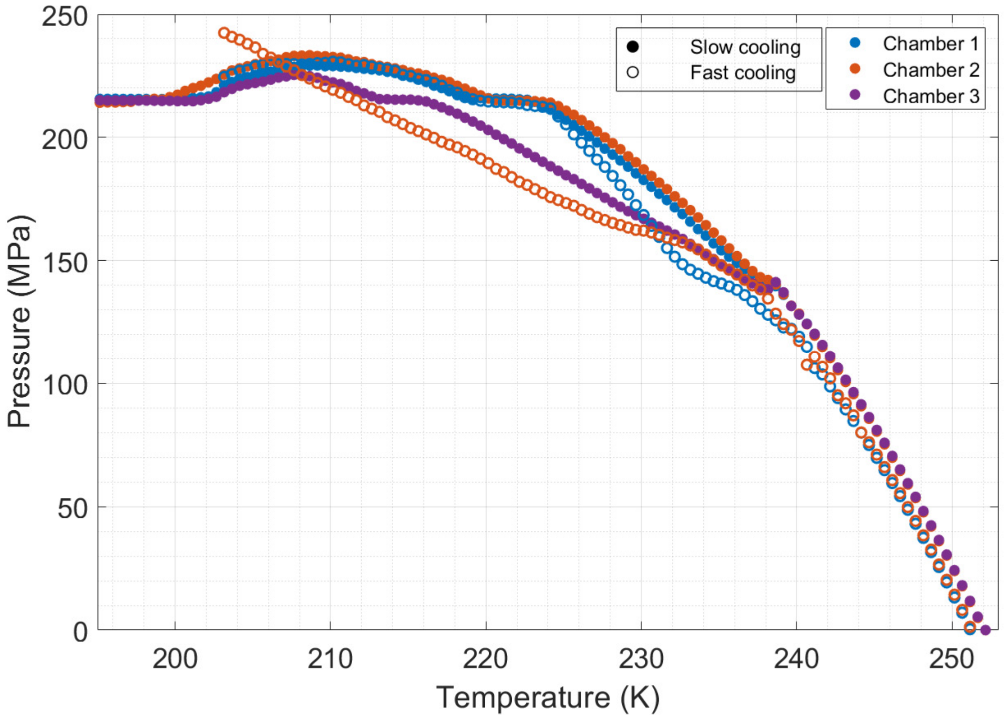

Supplementary Figure 4. All individual P-T curves recorded for NaCl.

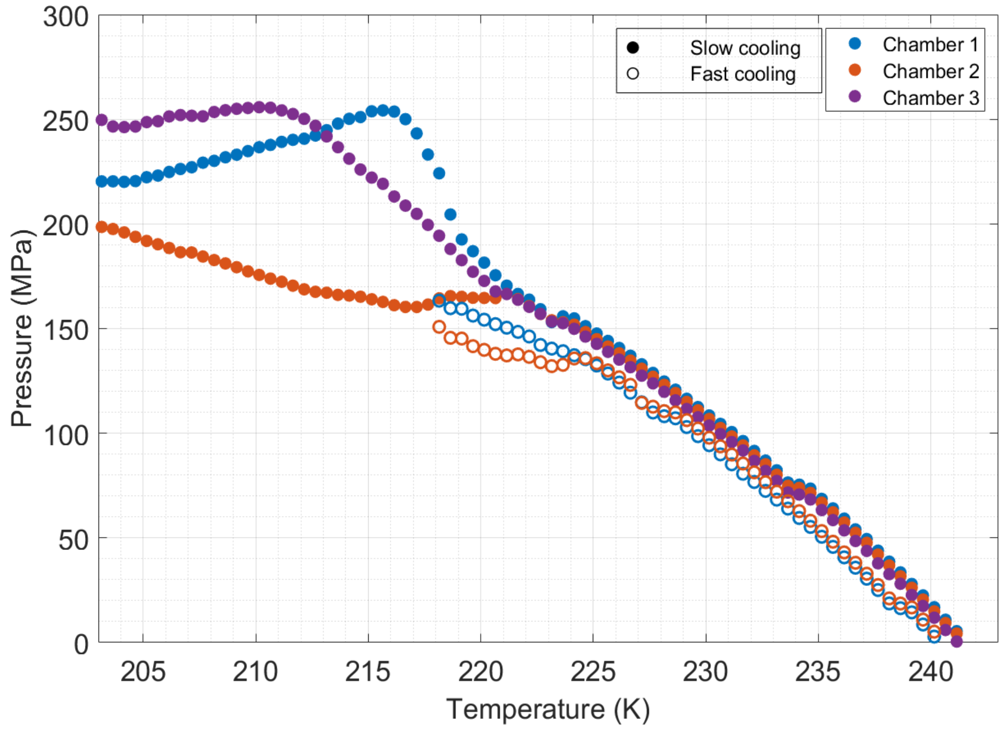

Supplementary Figure 5. All individual P-T curves recorded for MgCl2.

Supplementary Note 4. Uncertainty analysis

Our uncertainty analysis employs the propagation of uncertainties for independent variables using the root sum squared (RSS) method, as detailed by Taylor[8] and JCGM 100:2008[9]. Data presented in Figure 2 was obtained by measuring pressure at each steady temperature for every solution, as described in the methods section. The uncertainties in Figure 2a result from propagating the experimental standard deviation and the systematic error of the pressure sensors, which have an accuracy (NLHR) of 0.15% of span BFSL according to their data sheet. Temperature measurements were recorded via a calibration-grade Fluke thermocouple, and given its minimal systematic uncertainty (0.05℃), its effect was deemed negligible in our analysis.

The determination of the cenotectic point coordinates integrate the aforementioned uncertainties, as well as errors arising from polynomial curve fitting of the data. The phase transition at the cenotectic point, characterized by a discontinuity in the slope of the P-T curve of the solution, is mathematically defined as the intersection of the fitted polynomials. Due to measured uncertainties, this intersection point becomes a region, highlighted in Figure S6.

To ascertain the uncertainty region of the cenotectic point, the total uncertainty of the two fitted polynomials intersecting at the cenotectic point was calculated at each temperature increment. These uncertainties are considered independent, thus the total uncertainty at each temperature is the quadrature sum of the individual uncertainties, as follows:

$$\sigma_{total} = \sqrt{SEM^2 + \left(t\sigma_{fitting}\right)^2 + \sigma_{device}^2} \tag{1}$$

where SEM is the experimental standard error of the mean, $\sigma_{fitting}$ is the uncertainty of the fitted polynomial, t is the t-distribution factor, and σ_{device} is the uncertainty of the pressure sensor.

In accordance with JCGM guidelines, SEM is calculated as:

$$SEM = \frac{\sigma}{\sqrt{N}} \tag{2}$$

Where σ is the standard deviation of the measured pressure data at each temperature, and N is the number of technical replicates in each experiment, which, in this case, was 3 per solution. The uncertainty of the fitted polynomials before and after the cenotectic point was calculated using MATLAB's internal fit uncertainty algorithm, as evaluated from the polyfit() and polyval() functions. The t-distribution factor, as per JCGM recommendations, is determined based on the degrees of

freedom in the system and the desired confidence level, set at 95.45% in this study. Therefore, with 3 sensors at each experiment, the t-distribution factor of 3.31 was used. Device uncertainty was derived from the NLHR accuracy specified in the ESI datasheet for the 0-4000 bar pressure sensors (<0.15% BFSL), assumed to be constant with temperature.

By computing the total uncertainty of the fitted polynomials, the intersection of their upper and lower bounds was identified, delineating the uncertainty zone of the cenotectic point in the P-T diagram. Thus, uncertainty in cenotectic pressure is reported as the maximum vertical difference between any two points in this uncertainty zone, and uncertainty in cenotectic temperature is reported as the maximum horizontal difference between any two points in the same.

Similarly, the uncertainty of the eutectic temperature is determined by the difference between the upper and lower bounds of the intersection of the final fitted polynomial with 0.1 MPa in the diagram. Table 1 presents the calculated uncertainties of the cenotectic and eutectic characteristics for each tested solution.

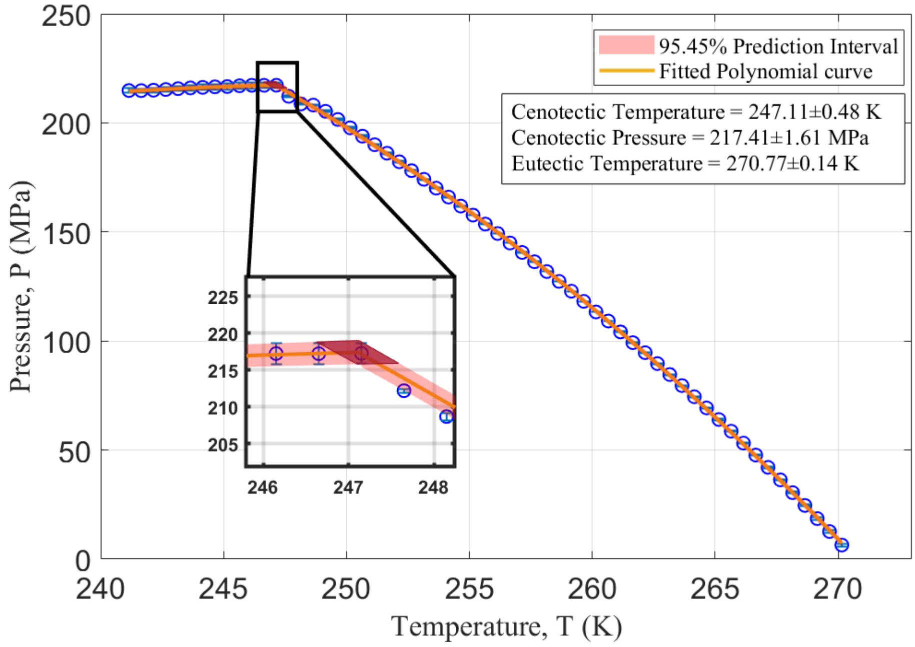

Supplementary Figure 6. The uncertainty analysis on the cenotectic point for Na_2CO_3 solution

Supplementary Note 5: Definition of the cenotectic point in the context of Gibbs' Phase Rule

In order to aid future study of the centotectic point, we provide in this note additional description of its definition in the context of Gibbs Phase Rule.

Per the definition in the main text, *the cenotectic is the invariant point occurring at the lowest temperature at which the liquid phase, for any value of concentration, pressure, or other thermodynamic forces acting on the system, remains in stable equilibrium.*

Gibbs Phase Rule offers some additional insight into the cenotectic point, by prescribing how many phases may be present at an any invariant point in a given system. For simple systems unaffected by modes of thermodynamic work other than mechanical (PV), thermal (TS), and chemical (muN) work, Gibbs Phase Rule gives: F = C – P + 2, wherein F is the number of intensive thermodynamic degrees of freedom, C is the number of chemical components in solution, and P is the number of phases present. Invariant points such as the cenotectic are defined F = 0 degrees of freedom, and as such, the number of phases present at the cenotectic P_K may be calculated as P = C + 2. For example, in the simple binary (C = 2) solutions investigated in this work, there will be P = 4 phases present at the cenotectic (i.e. the brine, ice-Ih, ice II/III, and the solute-bearing solid phase).

If the system *is* under the influence of non-simple forms of thermodynamic work, such as electrical work (EP) or magnetic work (BM), the Generalized Gibbs Phase Rule must be applied instead: F = W – P + 1, wherein W is the number of *independent* thermodynamic conjugate variable pairs (i.e. modes of thermodynamic work) contributing to the free energy of the system. Thus, the number of phases present at the cenotectic in a non-simple system is given by P = W + 1. For example, in a binary solution also under the influence of electrical work (i.e. exposed to an electrical field), W = (PV, TS, MuN, EP) = 4, and there will thus be P = 5 phases present at the cenotectic.

As such, a more rigorous mathematical definition of the cenotectic temperature specifically may be provided in terms of the Generalized Gibbs Phase Rule:

For any system affected by *W* independent modes of thermodynamic work, there exists some set of invariant point temperatures *IPT* at which *W+1* phases coexist, and the cenotectic temperature T_κ is the minimum of this set:

$$T_\kappa = \min\{IPT\}$$

Given that W+1 phases can only coexist at invariant points for which there are 0 intensive thermodynamic degrees of freedom, the cenotectic pressure (or the value of any other intensive variable at the cenotectic point) may be defined circularly as the

pressure corresponding to the cenotectic temperature, i.e., the point is fully prescribed by its temperature.

Supplementary References:

1. Pascual, M. R., Trambitas, D., Calvo, E. S., Kramer, H. & Witkamp, G. J. Determination of the eutectic solubility lines of the ternary system NaHCO3-Na2CO3-H2O. *Chemical Engineering Research and Design* **88**, (2010).

2. Li, G., Hwang, Y., Radermacher, R. & Chun, H. H. Review of cold storage materials for subzero applications. *Energy* vol. 51 Preprint at https://doi.org/10.1016/j.energy.2012.12.002 (2013).

3. Pillay, V. *et al.* MgSO4 + H2O system at eutectic conditions and thermodynamic solubility products of MgSO4·12H2O(s) and MgSO4·7H2O(s). *J Chem Eng Data* **50**, (2005).

4. Yuan, L. *et al.* Precise Urea/Water Eutectic Composition by Temperature-Resolved Second Harmonic Generation. *Chem Eng Technol* **39**, (2016).

5. González Díaz, C. *et al.* Thermal conductivity measurements of macroscopic frozen salt ice analogues of Jovian icy moons in support of the planned JUICE mission. *Mon Not R Astron Soc* **510**, (2022).

6. Journaux, B. *et al.* On the identification of hyperhydrated sodium chloride hydrates, stable at icy moon conditions. *Proc Natl Acad Sci U S A* **120**, (2023).

7. Ketcham, S. A., Minsk, L. D., Blackburn, R. R. & Fleege, E. J. Anti-icing: Lower the cost of safer roads. *Public Works* **128**, (1997).

8. Taylor, J. R. An Introduction to Error Analysis. *Journal of the Acoustical Society of America* **101**, (1997).

9. JCGM 100. JCGM 100:2008 - Evaluation of measurement data - Guide to the expression of uncertainty in measurement. *International Organization for Standardization Geneva ISBN* **50**, (2008).
